# Mathematical and Negative Information Are Similarly Processed: Pupil Dilation as an Indicator

**DOI:** 10.3390/jintelligence10040079

**Published:** 2022-10-03

**Authors:** Lilach Layzer Yavin, Adi Shechter, Orly Rubinsten

**Affiliations:** 1 Department of Learning Disabilities, Faculty of Education, University of Haifa, Haifa 3498838, Israel; 2 Edmond J. Safra Brain Research Center for the Study of Learning Disabilities, University of Haifa, Mount Carmel, Haifa 3498838, Israel

**Keywords:** mathematics, emotional valence, pupil dilation, semantical processing, cognitive effort

## Abstract

Background: Emotional perception of math-related information can affect an individual’s attitude and professional choices, especially in the area of science, technology, engineering, and math (STEM) professions. Method: The study compared the processing of math-related words, words with negative emotional valence, and words with neutral valence, using the physiological measure of pupil dilation on a random sample of 30 adults. Pupil responses were examined during a lexical decision task (LDT). We sought to show that exposure to math-related stimuli would cause arousal of the sympathetic system leading to an increase in pupil dilation, similar to that caused by exposure to negative stimuli. Results: pupillary responses were sensitive to words with emotional valence; exposure to math-related words led to increased pupil dilation compared to neutral words; exposure to words with negative valence led to increased pupil dilation compared to neutral words; exposure to math-related words and words with negative valence led to similar pupil dilation. The study concludes math-related textual stimuli lead to increased pupil dilation, similar to negative affective valence textual stimuli. Conclusion: These findings create new possibilities for studying the cognitive and emotional effort required to process math-related information using pupillary response, with implications for researchers, educators, and leaders in the field.

## 1. Introduction

Mathematics, engineering, and other technological fields are a central part of the digital age and are considered key factors in personal and economic success (Gravemeijer et al. 2017; Maass et al. 2019), yet some people avoid these fields, because they have negative emotions about math (Hembree 1990; Daker et al. 2021; Quintero et al. 2022). These negative emotions may affect their math achievements (Hannula 2019; Tuohilampi et al. 2014; Barroso et al. 2021) and, in turn, their life choices. To facilitate success in the technological age and understand how to steer individuals towards science, technology, engineering, and math (STEM) careers, it would be helpful to understand the negative emotions associated with numerical information.

Commonly used experimental paradigms exploring the relations between emotions and cognitive processes typically present different types of emotional words (i.e., threatening or neutral words) to participants (Silk et al. 2009; Siegle et al. 2011; Abado et al. 2020). According to cognitive theory, an individual’s perception of a word’s valence (e.g., negative emotional words, Palazova et al. 2013; food-related words, Gilon Mann et al. 2018) depends on the way the individual processes and emotionally interprets threatening information (Abado et al. 2020) as opposed to neutral information. In this theory, then, words are understood as powerful stimuli for examining emotions.

Previous studies in the field of numerical cognition and math education have used behavioral (e.g., questionnaires, Tuohilampi et al. 2014; Lewis 2013; Orbach et al. 2019) or physiological (e.g., blood pressure, Hunt et al. 2017; salivary cortisol, Mattarella-Micke et al. 2011) measurements to assess emotional valence linked with mathematics. Behavioral measurements may be biased, however, as most emotional processing happens subconsciously (Beck and Egger 2018). Measuring the physiological and biological indices that represent the foundations of behavior (Fritz et al. 2019) may provide a more objective and precise account of cognition. One such measure of emotional arousal is pupil size (Partala and Surakka 2003; Bradley et al. 2008; Kinner et al. 2017), a simple and non-invasive physiological tool. Specifically, pupil size measurement utilizes a screen and a camera, does not require specially attached devices and requires only a quick and automatic calibration process. Pupillometry has been found to be a useful, sensitive, and reliable measure of cognitive effort (Hess and Polt 1964; Kahneman 1973; Alnæs et al. 2014; Shechter and Share 2021) and emotional effort (Sirois and Brisson 2014; Peinkhofer et al. 2019).

In this study, we used a physiological measure; using math-related words as stimuli, we examined emotional perception by measuring pupil dilation. We aimed to shed light on attitudes towards math in an accurate and autonomous manner.

### 1.1. Review of the Literature

#### 1.1.1. Emotional Perception of Math-Related Information

Beliefs, emotions, and other affective phenomena are important elements of math perception (Hannula 2019). The emotional perception of math information is tied to abilities and accomplishments in math during the school years and also with the later decision to follow a STEM career (Hannula 2019; Seo et al. 2019). Negative emotional perception of math information can lead to an avoidance of learning math or avoidance of engaging in STEM altogether (Seo et al. 2019; Chang and Beilock 2016).

Previous studies have found that as early as primary school (Rapoport et al. 2016), some students show a decrease in positive emotions toward mathematics (Tuohilampi et al. 2014). Throughout the school years up until high school, an increasing number of students perceive mathematics as negative (Tuohilampi et al. 2014). Examining emotional perception of math-related information is important if we are to understand the source of avoidance, apprehension, and lack of motivation to deal with math information in any form (Lewis 2013).

#### 1.1.2. Ways to Measure Emotional Perception of Math-Related Information

Emotional perception of math-related information can be measured in a number of ways; common methods are behavioral measurements and physiological measurements. Behavioral measurements are generally based on self-reports, but these tend to be overly age-restricted or lack adequate statistics to support their validity (Carey et al. 2017). Self-report metrics also assess only cognitive and emotional dimensions. They do not evaluate physiological and biological levels, but these are important to determine the precise effect of emotions on the psychological and academic state of the individual (Fritz et al. 2019). Furthermore, categorizing individuals based on their math performance level using self-report scales fails to provide information on the real impact of a condition in daily and academic life (Fritz et al. 2019). Finally, self-report metrics lack the ability to assess emotions in real time (Orbach et al. 2019). In contrast, physiological measurements can provide information about the real impact during the performance of math-related tasks in real time.

Previous studies have examined emotional perception of math-related information using several types of physiological metrics, including: blood pressure (Hunt et al. 2017), salivary cortisol (Mattarella-Micke et al. 2011), skin conductance and heart rate (Qu et al. 2020), and amplitude in frontal and parietal areas using EEG (Núñez-Peña and Suárez-Pellicioni 2015) and MRI (Lyons and Beilock 2012).

One physiological measure found sensitive and applicable for examining emotional perception in previous studies is the measurement of pupil size (Peinkhofer et al. 2019; Silk et al. 2009). The measurement of systematic changes in pupil size is a non-invasive method for obtaining information on neurophysiological processes. Pupil size has been shown to be a valid and reliable index of emotional arousal (Peinkhofer et al. 2019) and word recognition (Geller et al. 2016), both of which can be helpful in identifying the emotional perception of math-related words. Yet, to the best of our knowledge, until now, no study has examined emotional perception specific to math information by measuring pupil size.

#### 1.1.3. Pupillometry as a Measurement of Emotional Perception

Pupil size is controlled by two parts of the autonomic nervous system: the sympathetic system controls the dilator muscles, and the parasympathetic system controls the sphincter muscles (Wetzel et al. 2016).

Cognitive effort leads to the simultaneous activation of the sympathetic system and the inhibition of the parasympathetic system (Bradley et al. 2008; Sirois and Brisson 2014; Finke et al. 2017). Pupil dilation has been proven as a sensitive and reliable indicator of cognitive effort (Kahneman 1973; Beatty 1982; Shechter and Share 2021) in a variety of domains, including emotions (Kinner et al. 2017; Peinkhofer et al. 2019; Cohen et al. 2015), cognitive workload (Unsworth and Robison 2017), auditory and visual attention capture (Marois et al. 2018; Mathôt et al. 2014), vigilance (Martin et al. 2022), and language (Shechter and Share 2021). More specifically, exposure to emotionally arousing stimuli (e.g., negative stimuli, Abado et al. 2020; negative, positive, neutral stimuli, Kuchinke et al. 2007) has been shown to cause an increase in pupil size (Peinkhofer et al. 2019).

Pupil dilation measurement has a high temporal resolution; thus, it provides a peripheral index of brain activation in response to a specific stimulus (Keil et al. 2018; Silk et al. 2009). A new approach to detecting temporal changes in pupil size using Bayesian analysis was recently proposed (Hershman et al. 2019; Hershman and Henik 2020). Examining temporal differences between conditions reveals different effects between time windows (Hershman et al. 2021). This type of analysis is useful for examining the time of occurrence of differences and how long they are maintained. In addition, temporal analysis can help avoid missing effects (Hershman and Henik 2020).

Many types of stimuli evoke an emotional response and thus may be suitable for examining emotions. For example, previous studies have examined emotional perception by using pupil response in exposure to auditory stimuli (Partala and Surakka 2003) and visual stimuli, including images (Cohen et al. 2015; Kinner et al. 2017) and words (Kuchinke et al. 2007; Silk et al. 2009). In our study, we explored the emotional perception of math-related words.

Thus, pupillometry is considered a sensitive physiological tool to assess a wide range of mental states, including emotional effort and cognitive workload. Since increased emotional effort and increased cognitive load are both expressed the same way, it is difficult to identify which one is the source; hence, we refer to the expression (pupil dilation) as well as the possible sources.

### 1.2. The Current Study

We focused on pupillary reaction to emotional stimulation, comparing pupillary responses to the processing of math-related words, words with negative emotional valence, and words with neutral valence. We exposed participants to these three types of word stimuli as part of a lexical decision task (LDT). Participants had to decide if the sequence of letters presented to them represented a real word or a nonword, i.e., a pseudoword (Hendrix and Sun 2021).

Previous studies have shown an increase in pupil dilation in response to stimuli with greater emotional intensity (Siegle et al. 2003; Silk et al. 2009; Kinner et al. 2017). Backed by existing research (Kuchinke et al. 2007; Peinkhofer et al. 2019), we examined whether there was a change in pupil dilation for negative stimuli (emotional arousal), neutral stimuli (no emotional arousal), and math-related stimuli. Specifically, we focused on the temporal changes in pupil dilation in exposure to math-related stimuli compared to the negative and neutral stimuli.

We expected to replicate previous findings showing increased pupil dilation during exposure to negative stimuli compared to neutral stimuli (Partala and Surakka 2003; Bradley et al. 2008; Kinner et al. 2017). More importantly, we hypothesized an increase in pupil dilation during exposure to math-related stimuli compared to neutral stimuli. We expected similar changes in pupil dilation during exposure to math-related stimuli compared to negative stimuli. We sought to show that exposure to math-related stimuli would cause arousal of the sympathetic system leading to an increase in pupil dilation, similar to that caused by exposure to negative stimuli (Peinkhofer et al. 2019).

We examined temporal changes in pupil dilation using Bayesian analysis. This approach is helpful in avoiding missing effects that appear for a short duration, as can be expected with averaging. It is also helpful in avoiding missing significant effects, as can be expected by analyzing over a narrow time window (Hershman et al. 2021).

## 2. Materials and Methods

### 2.1. Participants

On the basis of a priori power analysis, using effect sizes from previous studies (Cohen et al. 2015; Shechter and Share 2021), we estimated that a sample size of 30 students was required to detect sensitive pupillary responses to word recognition (Shechter and Share 2021) and emotional arousal (Cohen et al. 2015). Accordingly, data were collected from a random sample of 30 students from Haifa University who received course credit or a 40 ILS per hour monetary payment (around $13) for their participation. The study was carried out following the recommendations of the ethics committee of the University of Haifa with written informed consent from all subjects. All subjects gave written informed consent following the Declaration of Helsinki. The ethics committee approved the protocol of the University of Haifa (No. 010/21, date of approval: 11 January 2021). Three participants were excluded from the study due to equipment failures. An additional participant was excluded when the participant did not meet the criterion of at least 70% correct responses for each condition.

All participants were right-handed native Hebrew speakers, with no reported past or present attention deficits or reading difficulties or math difficulties, with normal or corrected vision, and all reported no general anxiety or math anxiety. The final sample comprised 26 participants (16 female, 10 male, mean age 27.68 years, SD = 6.19).

As part of an initial general assessment, participants were asked to fill out the short Mathematics Anxiety Rating Scale Questionnaire (sMARS; Alexander and Martray 1989) and the State-Trait Anxiety Inventory (STAI; Spielberger et al. 1983). In addition, to test their mathematical abilities, participants answered the Woodcock-Johnson III Test of Achievement; WJ-III (Woodcock et al. 2001). To define participants with anxiety (STAI; Spielberger et al. 1983), we set the cut-off scores for high state anxiety to >51 and for trait anxiety to >53 (Zsido et al. 2020; Spielberger et al. 1970).

### 2.2. Experimental Task: Pupillary Response to Math-Related Words during a Lexical Decision Task

#### 2.2.1. Stimuli

The experimental task included three types of word stimuli: 40 math-related words, 40 words with neutral valence, and 40 words with negative valence, all selected from a numerical-affective word database (Daches Cohen et al. 2021). Each word in the database was evaluated by a random sample of 290 adults (186 females); the sample evaluated the emotional valence of each word on a 5-point Likert scale. Math-related words specifically were measured on a 3-point scale for their relation to the field of mathematics (Daches Cohen et al. 2021). Daches Cohen et al. (2021) reported an insignificant difference in word-type frequency (math-related words, words with negative valance, and words with neutral valence).

One hundred and twenty pseudowords were formed from the letters of the three types of word stimuli (see Table 1). The pseudowords were created by scrambling the letters considering the preservation of the five word-ending letters (ך,ם,ן,ף,ץ) in the Hebrew language and preserving their position (Shechter and Share 2021).

There are a number of major non-cognitive caveats to using pupillary response as an indicator of emotional state. One is the pupillary light reflex whereby in response to sudden luminance, the pupil can quickly constrict to protect the eye (Ellis 1981). Another is the accommodative reflex, whereby the pupil constricts when the individual changes focus from looking at a faraway object to a near object, and when the individual’s eyes converge, such as when looking at the tip of the nose. In our experiment, the participants’ eyes and the screen were kept 57 cm apart using a head-rest, so there were no changes in the distance of the gaze. We also added a pre-trial stimulus of approximately the same shape and lighting as the actual stimuli, thus neutralizing the luminance change created by their appearance.

The stimuli word database used in the study contained pairs of equal-length words from the three types of stimuli (M = 6.225, SD = 1.83). To verify comprehension of the words, only the correct answers were included in the analysis. We examined the length of words for each group per subject. To ensure the length of the stimulus did not affect the experimental results, we performed a one-way ANOVA with word groups as the within-subject factor and word length as the independent variable. The effect of word length was found insignificant: F (2,50) = 3.32. *p* = .06, ηp2 = .12, BF10 = 1.90 (Table 2).

#### 2.2.2. Procedure

The task was divided into four blocks. Each block contained 10 math-related words, 10 neutral words, 10 words with negative valence, and 30 pseudowords (i.e., 60 stimuli per block). At the beginning of each block, instructions were displayed asking participants to read the words silently (without pronouncing them) and then, after a fixation screen had replaced the stimulus, to press the left mouse button for ‘word’ or the right mouse button for ‘nonword’ for each textual stimulus displayed on the computer screen. This type of task is called a lexical decision task (LDT).

**Figure 1:**
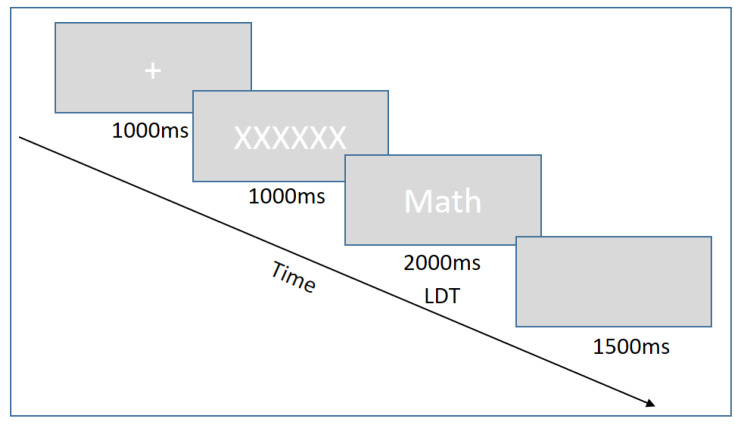
Lexical Decision Trial Design. A row of Xs is presented before the stimuli to avoid luminance confounding.

As shown in Figure 1, each trial began with a central cross (“+”) presented for 1000 milliseconds (ms) with a grey fixation screen (Hershman and Henik 2020; Kuchinke et al. 2007; Haro et al. 2017; Shechter and Share 2021). The fixation screen then presented a string containing a number of Xs equal to the number of characters in the upcoming letter string (Shechter and Share 2021; Moyes et al. 2019). The fixation was maintained for 1000 ms followed immediately by the letter string to minimize the effect of the pupillary light reflex. Each stimulus appeared on the screen for 2000 ms (Hershman and Henik 2020). The trial ended with a blank screen displayed for 1500 ms (Hershman and Henik 2020; Shechter and Share 2021).

#### 2.2.3. Data Analysis

##### Pupil Data Analysis

The baseline pupil diameter values were determined by averaging pupil size from 400 ms before the stimulus onset. Pupil data were processed using CHAP software (Hershman et al. 2019). The software automatically detects blinks, removes them, and performs interpolations to fill missing data. It then aligns time courses with the onset of the LDT stimulus and divides the result by the baseline value. Trials with 30% or more missing pupil values (pre-interpolation) were removed. For each condition, we required at least 70% correct answers for analysis. Incorrect responses and missing responses were excluded. In addition, Z scores were calculated and used to omit outliers (trials with Z scores exceeding 2.5) from further analyses. The Bayesian analysis showed differences between variables when BF_10_ > 3, and similarities between variables when BF_10_ < $\frac{1}{3}$.

##### Response Time and Accuracy Analyses

Response times were averaged within participants for correct answers for each of the three experimental conditions (three word types). Response times greater than two standard deviations above or below the participant mean were considered missing and excluded.

Lexical Decision Task Accuracy. For LDT accuracy, we observed a significant effect for group, F(2,50) = 25.73, *p* < .001, ηp2 = .51, BF10 = 853,773.01. Post hoc comparisons using *t-* tests revealed a significant difference, *t*(25) = −6.85, *p* < .001, between the response accuracy for math-related words (M = 86.70%, SD = 8.62) and neutral words (M = 96.05%, SD = 4.42). There was also a significant difference, *t*(25) = 3.74, *p* = .001, between the response accuracy for neutral words and words with negative valence (M = 93.36%, SD = 5.91). Finally, post hoc comparisons revealed a significant difference, *t*(25) = −3.83, *p* = .001, between the response accuracy for math-related words and words with negative valence (Table 2).

#### 2.2.4. Recording and Apparatus

Pupillometry data were obtained using a video-based eye tracker (Eyelink-1000 plus, SR Research, Kanata, ON, Canada) with a sampling rate of 1000 Hz. The LTD was built and presented using EyeLink’s Experiment Builder software. Participants’ eyes were 57 cm from a 24-inch LCD monitor (XL24II monitor, BenQ, Taipei, Taiwan; Quadro K620 graphics card, NVIDIA, Santa Clara, CA, USA) with 1024- × 768-pixel resolution and a refresh rate of 60 Hz. Each block was preceded by calibration and validation to ensure reliable pupil-size data. To maintain an accurate measure of pupil size before, during, and after the visual stimulation and to avoid contamination by saccadic eye movements, participants were instructed to keep their eyes focused on the screen and avoid shifting their gaze throughout the session. To avoid extreme luminance changes, the same white text (RGB values = 255, 255, 255) on a gray background (RGB values = 128, 128, 128) was used for all stimuli.

## 3. Results

### 3.1. Lexical Decision Task

#### 3.1.1. Behavioral Results

Response times and accuracy in LDT were analyzed using a one-way repeated-measures analysis of variance (ANOVA) with word groups (math-related words, neutral words, words with negative valence) as within-subject factors.

Response Time. For response time, we observed a significant effect for word group, F(2,50) = 12.08, *p* < .001, ηp2 = .33, BF10 = 389.99. Follow up *t*-tests revealed a significant difference, t(25) = 3.81, *p* = .001, between the response time for math-related words (M = 690.25, SD = 104.00) and neutral words (M = 661.36, SD = 117.51). They also showed significant differences, t(25) = 4.12, *p* < .001, between the response time for math-related words and words with negative valence (M = 657.43, SD = 117.82). There were no significant differences between the response time for words with negative valence and neutral words, t(25) = .64, *p* = .53.

#### 3.1.2. Results for Pupil Dilation

**Figure 2:**
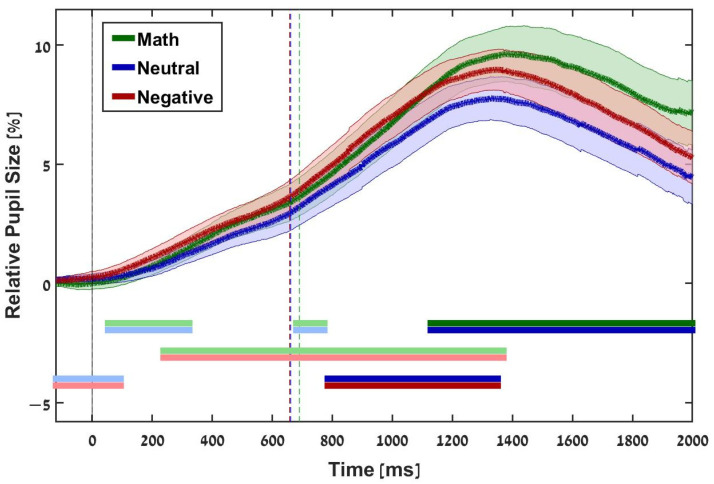
Relative Changes in Pupil Size for Three Conditions from Stimulus Onset (Time 0; black, dashed vertical line) to Trial Onset. Each colored, dashed vertical line marks the mean response time to the LDT task for the given condition. For each condition, the line curves represent changes in pupil dilation as a function of time. The shaded areas depict standard errors of the mean. The dark horizontal lines represent meaningful comparisons (BF_10_ > 3) for each contrast (e.g., the top green and blue lines indicate meaningful differences in pupil response to math-related words and words with neutral valence). The bright horizontal lines represent meaningful similarities (BF_10_ < $\frac{1}{3}$) for each contrast (evidence of the null hypothesis).

Recall that we considered correct answers only. Moreover, the pseudowords are not considered here, as they only serve the LDT functionally. Findings for mean relative changes of pupil dilation in each word condition are presented in Figure 2. Significant differences are represented by dark horizontal lines (e.g., the top two horizontal lines present significant differences between math-related words and neutral words).

Our analysis indicated significant differences between words with neutral valence and negative valence and math-related words and similarities between words with negative valence and math-related words. Specifically, the differences between words with negative valence (dark red line) and words with neutral valence (dark blue line) appeared at about 780 ms after the stimulus onset. These differences stayed for about 570 ms (until about 1350 ms after the stimulus onset). The differences between math-related words (dark green line) and words with neutral valence (dark blue line) appeared at about 1120 ms after the stimulus onset and stayed for about 880 ms (until about 2000 ms after the stimulus onset, the end of the trial). Similarities (evidence for the alternative hypothesis that two conditions are not the same) between math-related words (bright green line) and words with negative valence (bright red line) appeared at about 230 ms after the stimulus onset and continued for about 1170 ms (until about 1400 ms after the stimulus onset). We also found similarities between math-related words (bright green) and words with neutral valence (bright blue). These similarities appeared about 50 ms after the stimulus onset, stayed until about 300 ms (i.e., lasting about 250 ms), and appeared again for 100 ms (about 670 ms after the onset until 770 ms after the onset). Similarities between neutral words (bright blue) and negative words (bright red) appeared from 50 ms before the stimulus onset until about 95 ms after the stimulus onset. The last two similarities reinforced the previous findings and thus are detailed in the discussion.

### 3.2. Summary of Results

The study’s results clearly show that the pupil dilation responses of our participants were sensitive to words with emotional valence. As anticipated, a similarity in pupil dilation in responses to math-related words and words with negative valence appeared for about 1170 ms. A significant difference between neutral words and math-related words appeared for about 880 ms. Earlier, a significant difference between neutral words and words with negative valence appeared for about 570 ms.

## 4. Discussion

It has long been known that varied types of cognitive effort, specifically emotional effort and workload processing effort, can be examined using various physiological metrics (Peinkhofer et al. 2019; van der Ploeg et al. 2017). One such metric is pupil size (Bradley et al. 2008; Silk et al. 2009). Building on previous work suggesting math-related information is linked to negative affective valence (Daches Cohen et al. 2021), we examined pupil size change in exposure to different types of textual stimuli, specifically, math-related words, words with negative valence, and neutral words. In this part, we discuss the expression (differences in pupil dilation) of responses to math-related words compared to the other two (neutral, negative words), as well as the underlying possible sources (emotional effort, workload effort).

The study had three central physiological findings. First, consistent with previous studies, when exposed to stimuli with negative valence, participants showed increased pupil dilation compared to exposure to stimuli with neutral valence (Kuchinke et al. 2007; Partala and Surakka 2003; Bradley et al. 2008). The finding is in line with previous research showing that high emotional effort activates the sympathetic system, causing an increase in pupil dilation (Peinkhofer et al. 2019). Second, exposure to math-related stimuli showed delayed and increased pupil dilation compared to neutral valence stimuli. These findings can also be explained by an activation of the sympathetic system, caused by negative emotional arousal following an exposure to math-related stimuli (Daches Cohen et al. 2021). Third, throughout the measurement time, pupil dilation was found to be similar for exposure to math-related stimuli and exposure to negative stimuli. This important finding indicates the tendency of math-related stimuli to cause arousal of the sympathetic system, similar to negative stimuli. These three findings confirm the research hypotheses and indicate the physiological impact of exposure to math-related stimuli compared to negative and neutral stimuli is revealed in pupil dilation. We found exposure to math-related stimuli caused more cognitive effort and increased pupil dilation than exposure to neutral stimuli; this was similar to the difference we found between exposure to negative and neutral valence stimuli in different time lines.

Behavioral findings indicated insignificant differences in response time in the lexical decision task between negative stimuli and neutral stimuli. However, a significant difference in response time was found between math-related stimuli and neutral stimuli, and math-related stimuli and negative stimuli.

The findings provide strong evidence that pupillary responses are sensitive to cognitive effort (Shechter and Share 2021) in exposure to both math-related information and negative information, and the two responses are similar. The cognitive effort can be explained as an increase in workload (Unsworth and Robison 2017) when confronted by math-related words because of the semantic association (Shang et al. 2021) of the words’ meaning, or it can be explained as high emotional arousal (Sirois and Brisson 2014; Peinkhofer et al. 2019) and increased emotional effort.

The similarity in pupil dilation for math-related information and negative information appeared for a long period. An interesting finding for pupil dilation was that the differences between negative information and neutral information ended at the same time as the differences between math-related information and negative information; this can be explained by a drop in cognitive effort caused by the exposure to negative information past this period.

The differences in pupil dilation in exposure to neutral information compared to math-related information were delayed relative to the differences in pupil dilation in exposure to negative information compared to neutral information. The delay may be the result of the semantic cognitive effort required to process math-related information. Specifically, an increase in workload effort can cause a delay in the response to math-related information.

In conclusion, exposure to both math-related words and words with negative valence caused an increase in pupil dilation, compared to words with neutral valence. The increase in pupil dilation can be explained by the cognitive effort, including emotional effort and semantic workload effort. The physiological and behavioral findings are consistent with the hypothesis that exposure to math-related information will be expressed in a manner similar to exposure to negative information.

The emotional perception of math-related information (Hannula 2019) and the effect of emotion on life decisions (Hannula 2019; Seo et al. 2019) are known to be important. There are many options for examining the emotional perception of math-related information, but most are based on self-reports (Carey et al. 2017). We advanced the field by using a physiological factor, pupil dilation, to examine the emotional perception of math-related information, while using math-related words with semantic value. We found math-related stimuli tended to be perceived as having negative valence. This finding deepens existing knowledge about emotional effort, semantic processing, math-related perception, and the connections between them. The similarity of the pupil dilation expression in exposure to math-related information and in exposure to negative information reinforces the importance of continuing to deepen the knowledge about the sources and the treatment of math-related emotional perception.

### Limitations

The study examined subjects without math anxiety. To have a basis for comparison and a broader understanding of the emotional perception of math-related information, we would want to compare subjects without math anxiety to those with math anxiety. This limitation may be resolved in future studies by broadening the sample.

## 5. Conclusions

The study illustrates the physiological aspect of the emotions about math-related information in general. Specifically, our sample of adults experienced an increase in pupil dilation in exposure to math-related information, and this was the same as the increase in dilation in exposure to negative information. This conclusion highlights the emotional and semantic load a typical individual has to deal with in math situations. The findings should attract the attention of policymakers and educators seeking to increase the appeal of STEM professions, especially given the emotions that arise during exposure to math-related information and their impact on personal choices.

## Figures and Tables

**Table 1:** Example of Stimuli used in the Lexical Decision Task.

Lexical Decision Task (LDT)			
	Word		Nonword
Word Type	English word	Hebrew word	Pseudoword
Math-related words	Estimate	Omdan (אומדן)	Doman (דומאן)
Words with negative valence	Missiles	Tilim (טילים)	Litim (ליטים)
Neutral words	Drawer	Megira (מגירה)	Remiga (רמיגה)

**Table 2:** Behavioral Data: Mean and SD Response Time, Accuracy, and Word Length for Correct Answers in Each Group.

	Response Time (ms)		Accuracy (%)		Word Length (Letters)	
Word type	M	SD	M	SD	M	SD
Math-related words	690.25	104.00	86.70	8.62	6.17	0.15
Words with negative valence	657.43	117.82	93.36	5.91	6.13	0.11
Neutral words	661.35	117.51	96.06	4.42	6.20	0.05

## Data Availability

Data are available from coauthor Orly Rubinsten, orly.rubinsten@gmail.com.
